# All Nonmetal Resistive Random Access Memory

**DOI:** 10.1038/s41598-019-42706-9

**Published:** 2019-04-16

**Authors:** Te Jui Yen, Andrei Gismatulin, Vladimir Volodin, Vladimir Gritsenko, Albert Chin

**Affiliations:** 1 Department of Electronics Engineering, National Chiao Tung University, Hsinchu, 300 Taiwan; 2 Rzhanov Institute of Semiconductor Physics, Siberian Branch, Russian Academy of Sciences, Novosibirsk, Russia; 3 Novosibirsk State University, Pirogova street, 2, Novosobirsk, 630090 Russia; 4 Novosibirsk State Technical University, K. Marx ave., 20, Novosibirsk, 630073 Russia

## Abstract

Traditional Resistive Random Access Memory (RRAM) is a metal-insulator-metal (MIM) structure, in which metal oxide is usually used as an insulator. The charge transport mechanism of traditional RRAM is attributed to a metallic filament inside the RRAM. In this paper, we demonstrated a novel RRAM device with no metal inside. The N^+^-Si/SiO_x_/P^+^-Si combination forms a N^+^IP^+^ diode structure that is different from traditional MIM RRAM. A large high-resistance/low-resistance window of 1.9 × 10^4^ was measured at room temperature. A favorable retention memory window of 1.2 × 10^3^ was attained for 10^4^ s at 85 °C. The charge transport mechanism of virgin, high- and low-resistance states can be well modeled by the single Shklovskii-Efros percolation mechanism rather than the charge transport in metallic filament. X-ray photoelectron spectroscopy demonstrated that the value of x in SiO_x_ was 0.62, which provided sufficient oxygen vacancies for set/reset RRAM functions.

## Introduction

Resistive Random Access Memory (RRAM)^1–23^ is the highly promising candidate for the next generation nonvolatile memory (NVM), because conventional charge-based memories, namely dynamic random access memory and flash memory, have too low capacitance after continuously downscaling into 1X-nm regimes. In addition, an RRAM array can be fabricated in the back end of line of a complementary metal-oxide-semiconductor circuit, which makes such device an excellent candidate for embedded NVM (eNVM) application. The typical write speed of RRAM device ranges from 100 ns to 1 μs, which is three-to-four orders of magnitude faster than flash memory. Such high-speed and process-compatible eNVM can enable hardware technologies such as artificial intelligence and neuromorphic computing^1,6–8^.

The charge transport mechanism of RRAM, however, is not fully understood, and it is generally attributed to charge transport in metallic filament because of its metal–insulator–metal (MIM) structure, where the insulator is usually formed by metal oxide–based dielectric. Previously we pioneered nonmetal GeO_x_ RRAM, but the metal electrodes used might have contributed to the charge transport mechanism^16–21^. In the paper, we report the all nonmetal RRAM that does not contain any metal in both the electrodes and dielectric insulator. The purpose of all nonmetal RRAM device is to provide a different charge transport mechanism rather than the charge transport in normal metallic filament. Relatively small device variation and tight distribution can be reached in similar GeO_x_ RRAM^16^ that are crucial for array design^17^. After forming the RRAM device under 6 V and 100 μA current compliance, a large resistance window of 1.9 × 10^4^ was measured at room temperature (RT), which decreases slightly to 8.7 × 10^3^ after 10^4^ s data retention. The set/reset charge transport for low- and high-resistance states (LRS and HRS), deduced from the measured current–voltage (*I–V*) characteristics, is attributed to the charge transport mechanism by Shklovskii-Efros (S-E) percolation model.

## Results

**Figure 1:**
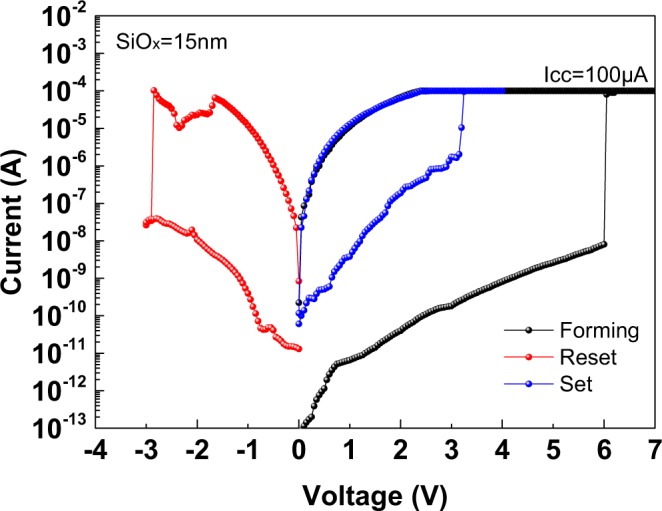
*I-V* characteristics of N^+^-Si/SiO_x_/P^+^-Si RRAM device under forming, set and reset operations.

Figure 1 depicts the measured *I-V* characteristics of an N^+^-Si/SiO_x_/P^+^-Si RRAM device. During the forming step, the device was first subjected to a 6 V and 100 μA compliance current stress to attain the LRS. The same device was reset into HRS after a negative voltage bias. Then, the device was set to LRS again under a positive voltage bias. However, the positive set voltage was lower than the forming voltage once the RRAM switching function was established.

**Figure 2:**
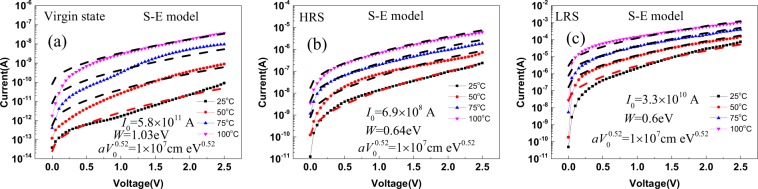
*I-V* dependences of (**a**) VS, (**b**) HRS and (**c**) LRS currents of N^+^-Si/SiO_x_/P^+^-Si RRAM and fitting curves of S-E model.

The charge transport mechanism is crucial for RRAM devices. To understand the charge transport mechanism in this completely nonmetal RRAM, we further analyzed the measured *I-V* curves at different temperatures. Figure 2(a–c) depict the measured and modeled *I-V* curves in the virgin state (VS), HRS and LRS conditions, respectively. All state the HRS and LRS currents adhere to the Shklovskii-Efros (S-E) percolation model:

1 $$I={I}_{0}\,\exp (-\frac{{W}_{e}-{(Ce\frac{U}{d}a{V}_{0}^{\gamma })}^{\frac{1}{1+\gamma }}}{kT}),$$

where *I*_0_, *W*_e_, *a*, *V*_0_, *C* and *ɣ* are the preexponential factor, percolation energy, space scale of fluctuations, energy fluctuation amplitude, numeric constant and it is equal to 0.25, critical index and it is equal to 0.9, respectively. The simulation by the S-E model gives reasonable model parameters to all resistance state (Fig. 2). The percolation energy decreases with decreasing resistance. Also, in the S-E model for LRS, the active contact area reduction of the charge involved in the transport is taken into account. The relation *a* × *V*_0_^0.52^ = 1 × 10^−7^ cm·eV^0.52^ does not change from resistance to resistance. This is due to the fact that with decreasing resistance increases space scale of fluctuations *a* but decreases energy fluctuation amplitude *V*_0_. In addition, it can be said that the S-E percolation model is applicable to the LRS case, then it can be assumed that the conducting channel is not continuous. Hence, the results demonstrate that the charge transport of the N^+^-Si/SiO_x_/P^+^-Si RRAM in VS, HRS and LRS are described by the S-E percolation model. For more details on other models and their inapplicability to HRS, see the ref.^24^.

**Figure 3:**
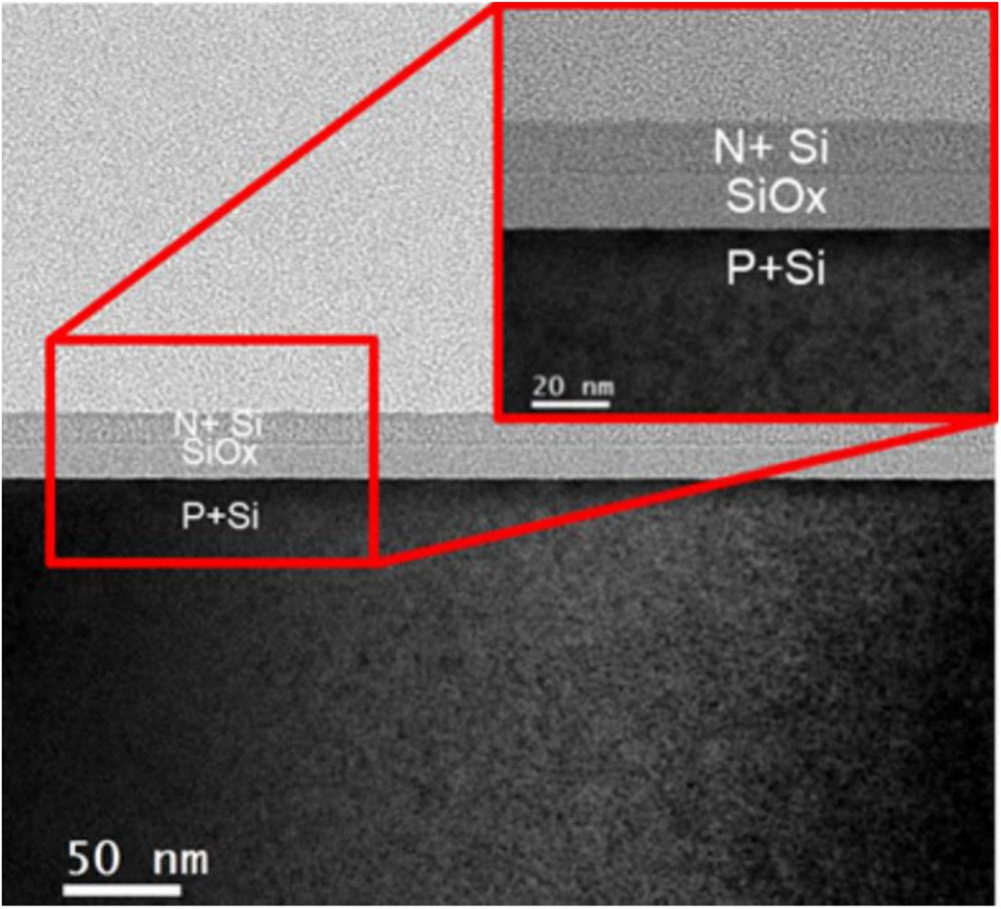
TEM image of N^+^-Si/SiO_x_/P^+^-Si RRAM devices.

**Figure 4:**
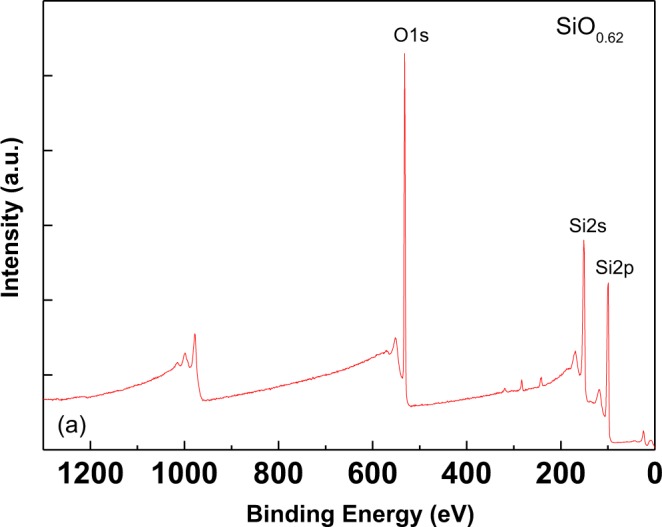
XPS spectrum of SiO_x_ layer.

To further understand the device characteristics, material analyses were performed. Figure 3 displays the cross-sectional transmission electron microscope (TEM) image of this RRAM device. As depicted, the RRAM device was fabricated directly on a P^+^-Si substrate, followed by a 15-nm thick SiO_x_ dielectric layer and a N^+^-Si top electrode. The SiO_x_ layer was further analyzed using X-ray photoelectron spectroscopy (XPS). The sample surface was pre-sputtered to ensure that the native oxide did not influence the measurements. Figure 4 displays the XPS spectrum. From the peaks of O1s, Si2s, and Si2p, the mole fraction x in SiO_x_ was determined to be 0.62. Because no metal or metallic ions were present in the whole RRAM device, metallic filaments were not formed^13–16^. In accordance with XPS experimental data certain fraction of vacancies exist in dielectric immediately after synthesis. The migration of oxygen vacancies plays an important role for current conduction.

**Figure 5:**
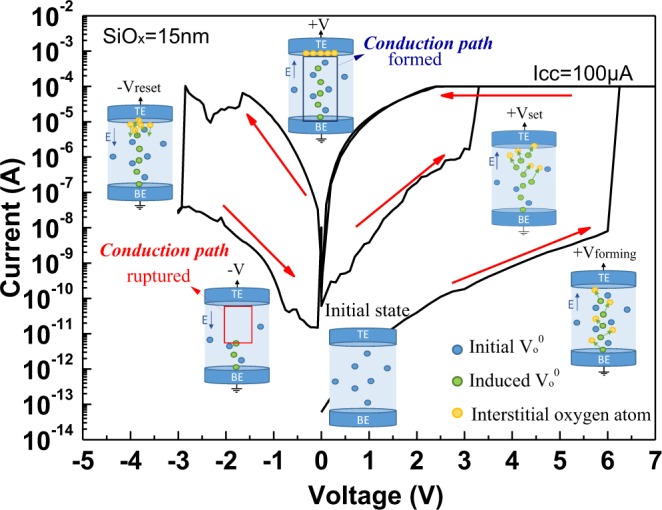
Potential microscopic conduction of N^+^-Si/SiO_x_/P^+^-Si RRAM device.

Figure 5 plots potential switching mechanisms. During the forming step, the current conducted through the initial V_o_^2+^ inside the SiO_x_ layer^14^. When the RRAM device was under sufficiently high positive voltage, soft breakdown in SiO_x_ occurred and disrupted the covalent bonds^25,26^, generating unbonded Si ions, O^2−^ and V_o_^2+^. In refs.^27,28^, it was shown that V_o_^2+^ and O^2−^ anti-Frenkel pairs are not stable and should recombine immediately. We assume, that after generation of anti-Frenkel pairs, electrons are redistributed to maintain charge neutrality and new oxygen vacancies (V_o_^0^) and interstitial oxygen atoms are formed^29^. Because the atomic size of O is significantly smaller than Si, the interstitial oxygen atoms and V_o_^0^ could migrate inside SiO_x_ under the applied electric field. At the end of the forming process, the interstitial oxygen atoms were attracted to the positive voltage and accumulated at the interface of top N^+^I junction. Once the conduction path was formed, electrons could transport through the V_o_^0^ creating the LRS current pass in the SiO_x_ layer. After application of a negative voltage, interstitial oxygen atoms moved away from the top N^+^I junction and recombined with V_o_^0^ to rupture the conduction path- the reset process. After a positive voltage was applied again, the set process behaved as the forming process to form a conduction path, but under a lower positive voltage than the forming voltage due to not all generating in forming process V_o_^0^ recombined in reset process.

**Figure 6:**
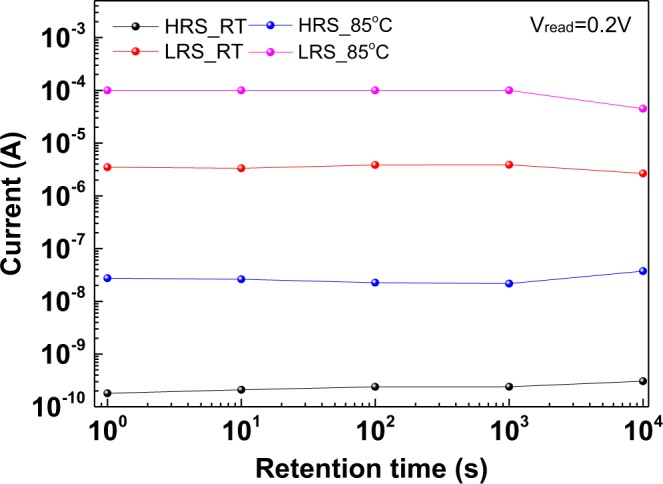
Retention characteristics of N^+^-Si/SiO_x_/P^+^-Si RRAM devices at RT and 85 °C.

Data retention is the necessary characteristics for NVM, and they are related to the nonvolatile behaviour and lifetime of an RRAM device. Figure 6 depicts the retention characteristics of the N^+^-Si/SiO_x_/P^+^-Si RRAM device. The completely nonmetal RRAM device could achieve favourable retention with a slight resistive window decay from 1.9 × 10^4^ to 8.7 × 10^3^ at RT and 3.6 × 10^3^ to 1.2 × 10^3^ at 85 °C after 10^4^ s retention.

## Conclusion

A completely nonmetal RRAM device was demonstrated for the first time. A large resistance window of 1.9 × 10^4^ at RT was measured. An excellent retention resistance window of 1.2 × 10^3^ was obtained for 10^4^ s retention at 85 °C. In addition, the charge transport of the N^+^-Si/SiO_x_/P^+^-Si RRAM in VS, HRS and LRS are described by the S-E percolation model. And the V_o_^0^ migration played an important role in the set/reset functions.

## Methods

The RRAM device was made on a highly doped P^+^-Si substrate with a resistance lower than 0.01 Ω per square, which was also used as a bottom electrode. After standard RCA clean, the native oxide on P^+^-Si wafer was removed by a dilute hydrofluoric (HF) acid (HF: H_2_O = 1:100) solution for 60 sec. Then, a 15-nm-thick SiO_x_ was deposited by reactive sputtering. The composition ratio inside the SiO_x_ was determined using XPS. Then, a 15-nm-thick amorphous N^+^-Si layer, was formed as the top junction electrode. The diameter of the fabricated device was 120 μm. The *I-V* characteristics was measured using an HP4155B parameter analyzer. The voltage was applied on the N^+^-Si (top electrode) side and P^+^-Si (bottom electrode) were grounded. The sweep rate is 0.5 V/s. A Thermo K-alpha system with an X-ray spot size of 400 μm was employed for XPS measurements. The cross-sectional image of the RRAM device was measured using a JEOL 2010F high-resolution TEM. The modeled data for HRS and LRS were fitted under positive and negative voltage bias, respectively.
